# Dilemmas of nomenclature: Web search analysis reveals European preferences in atopic skin diseases

**DOI:** 10.1002/clt2.12355

**Published:** 2024-04-12

**Authors:** Hannah Wecker, Stefanie Ziehfreund, Sebastian Sitaru, Emma K. Johansson, Jesper Elberling, Anaïs Doll, Electra Nicolaidou, Emanuele Scala, Michael J. Boffa, Lea Schmidt, Mariusz Sikora, Tiago Torres, Pavel V. Chernyshov, Alexander Zink

**Affiliations:** ^1^ Department of Dermatology and Allergy Technical University of Munich TUM School of Medicine and Health Munich Germany; ^2^ Division of Dermatology and Venereology Department of Medicine Solna Karolinska Institutet Stockholm Sweden; ^3^ Department of Dermatology and Venereology Karolinska University Hospital Stockholm Sweden; ^4^ Depart of Dermatology and Allergy Herlev and Gentofte Hospital Copenhagen Denmark; ^5^ Department of Clinical Medicine University of Copenhagen Copenhagen Denmark; ^6^ 1st Department of Dermatology and Venereology Medical School National and Kapodistrian University of Athens Athens Greece; ^7^ Department of Dermatology and Venereology Medical Center ‐ University of Freiburg Faculty of Medicine University of Freiburg Freiburg Germany; ^8^ Division of Dermatology and Venereology Department of Medicine Solna and Center for Molecular Medicine Karolinska Institutet Stockholm Sweden; ^9^ Department of Dermatology Mater Dei Hospital Msida Malta; ^10^ National Institute of Geriatrics, Rheumatology and Rehabilitation Warsaw Poland; ^11^ Department of Dermatology Centro Hospitalar Universitário de Santo António University of Porto Porto Portugal; ^12^ Department of Dermatology and Venereology National Medical University Kiev Ukraine

To the editor,

Atopic dermatitis (AD) or atopic eczema (AE) is a complex chronic inflammatory skin disease with a high prevalence and disease burden.^1^ The nomenclature for this condition has long been the subject of controversial debate within the medical community and even among global experts.^2^, ^3^ However, the terminology used not only affects experts, daily clinical practice, and research but especially patients and the general public in terms of their understanding and access to disease‐related information.^2^, ^3^, ^4^, ^5^ Given the potential of crowdsourced internet data,^6^ this study aimed to investigate the use of ‘atopic dermatitis’, ‘atopic eczema’, and their lay terms in internet searches and the content of these searches across 21 European countries in their respective main language.

A total of 71,620,240 AD‐related searches, 33,913,480 AE‐related searches, and 136,405,350 searches to the respective lay terms were identified across European countries between 02/2019 and 01/2023 using Google Ads Keyword Planner. The top 20 keywords for each country and search term were translated into English and inductively classified into 9 categories: *age group*, *causes*, *comorbidities*, *general* information, *localisation*, *other disease*, *others*, *symptoms*, and *treatment*. Subcategories were formed for recurring keywords, for example, different body localisations. For cross‐country comparison, the monthly number of web searches per 100,000 inhabitants was calculated. For detailed methodology, see the Appendix.

Most European countries (*n* = 11) had the highest median number of web searches per 100,000 inhabitants for AD‐related lay terms, followed by AD (*n* = 8) and AE (*n* = 2, Figure 1). Analysis revealed common search themes across European countries, including *general* disease information, *age groups*, *localisations*, and *treatment*, with slight variations between countries (Figure 2A). The lay term's keywords were often about other diseases. Depending on the search terms, internet queries in some categories focused on different subcategories (Figure 2B). For example, when using the lay term, more countries searched for *(natural) remedies* and anogenital localisations, and only AD‐related searches included searches for *animals*. However, there were also similarities between the search terms, with *face*, *hands*, and *scalp* being the most frequently searched localisations. *Age‐*related internet searches concerned primarily babies and children, whereas in Austria and Germany, adults were the only search subjects. Search content for lay terms appeared less differentiated than for the other search terms.

**FIGURE 1:**
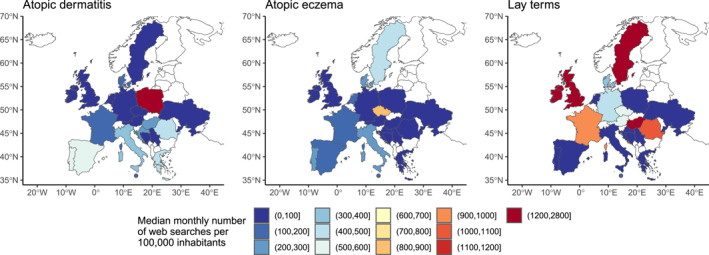
Median monthly number of web searches per 100,000 inhabitants for the search terms ‘atopic dermatitis’, ‘atopic eczema’, and the respective lay terms across the 21 European countries under study between February 2019 and January 2023. Malta is represented as an enlarged dot for enhanced visualisation.

**FIGURE 2:**
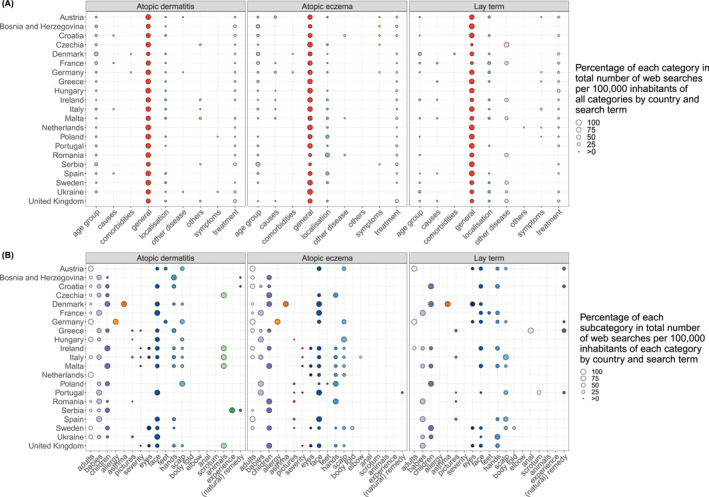
Percentage of each category (A) and subcategory (B) in the total number of web searches per 100,000 inhabitants by country and search term. The categories and subcategories were formed inductively from the top 20 keywords for each country and search term ‘atopic dermatitis’, ‘atopic eczema’, and the respective lay terms. Each category is represented by a distinct colour, with subcategories kept in similar shades to their respective categories.

Consistent with previous research, both a review study and a global crowdsourced approach found that the term AD was used more frequently than AE.^3^, ^4^ However, country‐specific lay terms received almost twice as many web searches and were favoured in most countries, suggesting the general population may not be interested in the academic debate about calling the disease AD or AE, but rather seeking general disease‐related information as the diagnosis may not yet be known.^4^ This is supported by less differentiated search content and a higher number of searches for other diseases. For example, the commonly used lay term ‘eczema’ lacks precision and can encompass other skin conditions such as seborrhoeic or nummular eczema, which present with eczematous lesions.^2^, ^3^ In addition, the varying topics and number of searches in different European countries may reflect country‐specific needs regarding AD in the population and should be considered when disseminating reliable and accurate online health information.^6^ Furthermore, the differences in content between, AD, AE and their lay terms may indicate confusion and the perception of different diseases in the population, which should be addressed in patient communication.^3^

The study highlights the preference for lay terms followed by AD and AE in Europe when searching for disease‐related information online. These findings advocate the standardisation of terms and language used in health information and patient communication, as well as the adaptation of information to the specific needs of each country.

## AUTHOR CONTRIBUTIONS

Hannah Wecker, Stefanie Ziehfreund, and Alexander Zink conceptualised the study and provided the first draft. Hannah Wecker performed the statistical analysis. Sebastian Sitaru, Emma K. Johansson, Jesper Elberling, Anaïs Doll, Electra Nicolaidou, Emanuele Scala, Michael J. Boffa, Lea Schmidt, Mariusz Sikora, Tiago Torres, and Pavel V. Chernyshov provided data and actively contributed to discussion of results and to the final manuscript draft. All authors contributed to the article and approved the submission.

## CONFLICT OF INTEREST STATEMENT

Hannah Wecker, Stefanie Ziehfreund, Sebastian Sitaru, Anaïs Doll, Electra Nicolaidou, Emanuele Scala, Michael J. Boffa, Lea Schmidt, Mariusz Sikora, and Pavel V. Chernyshov have no conflicts of interest to declare. Emma K. Johansson received speaker honoraria and/or been a consultant for AbbVie, ACO, Almirall, LEO Pharma, Novartis, Pfizer, Sanofi‐Genzyme, and the Swedish Asthma and Allergy Association. Jesper Elberling has been at the Advisory Board and/or received speaker's honoraria and/or support for meetings and travel from/of the following companies: Pfizer, Sanofi, Leo Pharma, Novartis, AstraZeneca, Almirall, AbbVie, Eli Lilly, Galderma, Takeda, CSL Vifor. Tiago Torres has received honoraria for acting as a consultant and/or as a speaker at events sponsored by AbbVie, Almirall, Amgen, Arena Pharmaceuticals, Biocad, Boehringer Ingelheim, Bristol‐Myers Squibb, Celgene, Eli Lilly, Janssen, Leo Pharma, MSD, Novartis, Pfizer, Samsung‐Bioepis, Sandoz, and Sanofi. Alexander Zink has been an advisor and/or received speaker's honoraria and/or received grants and/or participated in clinical trials from/of the following companies: AbbVie, ALK Abello, Almirall, Amgen, Beiersdorf Dermo Medical, Bencard Allergie, BMS, Celgene, Eli Lilly, GSK, Incyte, Janssen Cilag, Leo Pharma, Miltenyi Biotec, MSD, Novartis, Pfizer, Sanofi‐Aventis, Takeda Pharma, Thermo Fisher Scientific Phadia, UCB.

## FUNDING INFORMATION

Technical University of Munich

## Supporting information

Supporting Information S1

## Data Availability

The data that support the findings of this study are available from the corresponding author, AZ, upon reasonable request.
