# Supplementary material for: Dilemmas of nomenclature: Web search analysis reveals European preferences in atopic skin diseases
Source: Clin Transl Allergy. 2024 Apr 12;14(4):e12355. doi: 10.1002/clt2.12355 (PMC11015053; doi:10.1002/clt2.12355)
Supplement: Supplementary file 1 — Supporting Information S1 [file CLT2-14-e12355-s001.pdf]

Appendix
Methods

The Google Ads Keyword Planner was used to obtain web search data from the search engine and its network partners in 21 European countries, including Austria, Bosnia and Herzegovina, Croatia, Czech Republic, Denmark, France, Germany, Greece, Hungary, Ireland, Italy, Malta, Netherlands, Poland, Portugal, Romania, Serbia, Spain, Sweden, Ukraine, and United Kingdom. The tool generates keywords and phrases along with estimated monthly web searches for the past 48 months, based on the entered search terms. In the present study, the search terms "atopic dermatitis", "atopic eczema", and their lay terms were used separately for each country in the main language provided by native speakers and/or experts in the field of dermatology (Table S1). The AI-based translator DeepL was used to translate the terms for Czechia. For Malta and Bosnia, English and Serbian respectively were used, as the main spoken languages were not available in the tool. In order to ensure a comprehensive representation, the European countries were selected based on the authors' network of experts and native speakers.
For cross-country comparisons, the monthly number of web searches per 100,000 inhabitants was calculated based on the population size of each country in 2019, as this information was available for all the countries studied in that year, according to the Statistical Office of the European Union.[1] Institutional review board approval and informed consent were not required for this study.

For the content analysis of web searches, the 20 most common keywords for the search terms "atopic dermatitis", "atopic eczema", and the respective lay terms of each country were inductively categorised into 9 categories based on whether they referred to *age group* (e.g., "atopic eczema children"), *causes* (e.g., "atopic dermatitis causes"), *comorbidities* (e.g., "asthma eczema"), *general* information (e.g., "atopic skin"), *localisation* (e.g., "neurodermitis eyes"), *other disease* (e.g., "nummular eczema"), *others* (e.g., "atopic dermatitis dog"), *symptoms* (e.g., "neurodermitis symptoms"), and *treatment* (e.g., "neurodermitis treatment"). Each keyword was classified into one category or a respective subcategory, as applicable (Table S2).

Data were analysed descriptively using R version 4.2.3. (R Core Team, 2021, Vienna, Austria).

---

[1] Statistical Office of the European Union (01.06.2022) Population on 1 January [TPS00001]. URL https://ec.europa.eu/eurostat/web/main/data/database [accessed on 29.03.2023].

Table S1 The search terms "atopic dermatitis", "atopic eczema" and the respective lay terms in the main spoken language of the 21 European countries under study.

Country	Atopic dermatitis	Atopic eczema	Lay terms
**Austria**	Atopische Dermatitis	Atopisches Ekzem	Neurodermitis
**Bosnia and Herzegovina†**	Atopijski dermatitis	Atopijski ekcem	Neurodermitis
**Croatia**	Atopijski dermatitis	Atopijski ekcem	kronični Lišaj, Neurodermitis
**Czechia**	atopická dermatitida, atopické dermatitidy	atopicky ekzém	dermatitida, Zánet kûze
**Denmark**	Atopisk dermatitis	Atopisk eksem	Børneeksem, astmaeksem
**France**	dermatite atopique	eczéma atopique	neurodermite, neurodermatite, eczéma
**Germany**	Atopische Dermatitis	Atopisches Ekzem	Neurodermitis
**Greece**	ατοπική δερματίτιδα	ατοπικό έκζεμα	νευροδερματίτιδα
**Hungary**	atópiás dermatitis	atópiás ekcéma	ekcéma
**Ireland**	atopic dermatitis	atopic eczema	eczema, dermatitis
**Italy**	dermatite atopica	eczema atopico	neurodermatite
**Malta†**	atopic dermatitis	atopic eczema	eczema, dermatitis
**Netherlands**	Atopische dermatitis	Atopisch eczeem	Neurodermitis, Neurodermatitis
**Poland**	Atopowe zapalenie skóry	Wyprysk atopowy	Nurodermit, neurodermitis
**Portugal**	Dermatite atópica	Eczema atópico	Neurodermite, neurodermatite, líquen simples crónico
**Romania**	Dermatita atopică	eczemă atopică	eczemă, dermatită
**Serbia**	Atopijski dermatitis	Atopijski ekcem	Neurodermitis
**Spain**	dermatitis atópica	eccema atópico	Neurodermatitis
**Sweden**	atopisk dermatit	atopiskt eksem	böjveckseksem, eksem
**Ukraine**	Атопічний дерматит	Атопічна екзема	Діатез, нейродерміт, екзема
**United Kingdom**	atopic dermatitis	atopic eczema	eczema, dermatitis

† For Bosnia and Herzegovina and Malta, Serbian and English were used, respectively.

Table S2 The inductively formed categories and subcategories for recurring topics of the top 20 keywords of the 21 European countries and web search terms “atopic dermatitis”, “atopic eczema” and respective lay terms.

**Categories**	**Subcategories**	**Definition**
Age group	Adults, babies, children	Keywords related to age, e.g., “atopic eczema children”
Causes	-	Keywords related to the cause of the condition, e.g., “atopic dermatitis causes”
Comorbidities	Allergy, asthma	Keywords related to comorbidities of the condition, e.g., “asthma eczema”
General	Pictures, severity	General information about the condition e.g., “atopic skin”, “atopic eczema”
Localisation	Eyes, face, feet, hands, scalp, body fold, elbow, anal, scrotum	Keywords related to specific localisations of the condition, e.g., “neurodermitis eyes”
Other disease	-	Keywords related to another disease, e.g., “nummular eczema”
Other	Animals, experience	Keywords which do not fit into the existing categories, e.g., “atopic dermatitis dog”
Symptoms	-	Keywords related to the symptoms of the condition, e.g., “neurodermitis symptoms”
Treatment	(Natural) remedy	Keywords related to the treatment of the condition, e.g., “neurodermitis treatment”
